# “When Will You Graduate?”—A Qualitative Study on Academic Procrastination Among Italian University Students

**DOI:** 10.3390/ijerph23030374

**Published:** 2026-03-16

**Authors:** Jacopo Postiglione, Elisabetta Fenizia, Santa Parrello, Massimiliano Sommantico

**Affiliations:** Department of Humanities, University of Naples Federico II, Via Porta di Massa 1, 80133 Naples, Italy; jacopo.postiglione@unina.it (J.P.); elisabetta.fenizia@unina.it (E.F.); parrello@unina.it (S.P.)

**Keywords:** academic procrastination, university students, young adults, developmental tasks, future concerns, social expectations, perfectionism, psychological well-being, qualitative study, thematic analysis

## Abstract

**Highlights:**

**Public health relevance—How does this work relate to a public health issue?**

- Academic procrastination is associated with poorer psychological well-being outcomes, including increased stress and anxiety among university students.
- Social expectations of perfection and future-related worries emerge as key determinants of students’ procrastination and mental health.

**Public health significance—Why is this work of significance to public health?**

- The present study highlights how culturally reinforced performance pressure can contribute to maladaptive coping strategies, negatively affecting university students’ well-being.
- Understanding procrastination as a socially shaped phenomenon can support more effective prevention approaches for students’ mental health.

**Public health implications—What are the key implications or messages for practitioners, policy makers and/or researchers in public health?**

- Interventions should address perfectionism and future uncertainty through supportive academic environments.
- Findings support policies and research focusing on systemic academic pressure, not only individual self-regulation, to reduce procrastination.

**Abstract:**

Background: In contemporary societies, the pursuit of performance and the experience of urgency emerge as dominant forces shaping individual lives. In this context, delaying behaviors assume particular significance, especially for university students, who are immersed in environments that seem to prioritize speed and efficiency as the main routes to adulthood. The pressure to be flawless and fast, coupled with uncertainty about the future, calls for reflection on procrastination, its impact on psychological well-being, and the role of educational institutions. This study explored university students’ opinions and experiences regarding academic procrastination. Methods: Ten focus groups were conducted with 89 students enrolled in a Bachelor’s degree program. All focus groups were recorded and transcribed verbatim. The corpus was analyzed using Thematic Analysis of Elementary Contexts and Reflexive Thematic Analysis. Results: The former, a cluster-based thematic procedure, identified seven clusters capturing both the organizational aspects of university life and the experience of being a student in contemporary society. The secondary thematic analysis further explored these dimensions, emphasizing themes such as social pressure and concerns about the future. Conclusions: Findings suggest that understanding the dynamics underlying procrastination can inform university policies attuned to young adults’ developmental needs and well-being.

## 1. Introduction

Dilatory behaviors, in both their adaptive and maladaptive forms, have long been studied across multiple disciplines. A bibliometric analysis conducted by Yan and Zhang [1] shows that scientific interest and publications on the topic have grown steadily since the 1990s, with a gradual linear increase since 2010. Among studies on procrastination, the tendency to delay in academic contexts has undoubtedly been one of the most extensively examined aspects in psychological research, emerging, due to its prevalence, as a phenomenon of epidemic proportions [2]. Indeed, several studies suggest that this type of procrastination affects at least 50% of students [3,4]. Moreover, it concerns graduates, university and high school students [5], “traditional” and “non-traditional” students [6], and is widespread in different cultural contexts [7,8].

Specifically, academic procrastination refers to the irrational tendency to delay the start or completion of academic tasks [9] and is the most well-known form of situational procrastination—i.e., the act of postponing in specific contexts or situations [10].

According to Sirois and Pychyl [11], procrastination is far more than a byproduct of inadequate time management; it reflects a behavioral response to tasks perceived as adverse. Such aversion generates negative emotions whose repair takes priority over the self-regulation necessary to achieve intended goals. While this may serve to repair mood in the short term, it exposes individuals to long-term negative consequences and increases the likelihood of entering vicious circles that reinforce maladaptive behavior [12,13].

Several studies have shown that academic procrastination has detrimental effects on students’ academic achievement, measured, for example, by GPA [14,15,16,17,18]. Furthermore, it has been observed that procrastination affects not only performance but also physical and psychological well-being [19]. For instance, research has shown that students’ procrastination is positively associated with psychological distress [20,21,22,23] and negatively associated with life satisfaction [21,24,25].

Evidence of the negative consequences of procrastination across different domains has led to widespread interest in studying its antecedents. In particular, research has focused primarily on personality characteristics and motivational or volitional dimensions, largely neglecting contextual factors that can either favor or inhibit the tendency to postpone tasks [26]. In recent years, however, there has been growing attention to the “situational perspective” [3], which offers a valuable contribution to understanding the complexity of the origins and manifestations of academic procrastination.

Attention to external factors appears particularly relevant in university contexts, which have been defined as “procrastination-friendly” environments [27]. Accordingly, Svartdal et al. [28] conducted an overview of the literature that allowed them to identify nine factors that seem to favor academic procrastination: a large degree of freedom in study situations, excessively long deadlines, aversion to the task, presence of temptations and distractions, limited information for effective self-control, little attention to training in study skills, lack of opportunities to develop self-efficacy, ineffective teamwork, and peer influence.

Other studies [29,30] identified additional characteristics, including anonymity at the university, low course situational interest in courses, inefficient exam organization that results in many requests at once, and a perception of non-recognition of student achievements. Findings from such studies highlight the university as a distinctive context in which the external regulation typical of previous educational stages is no longer present. Unlike secondary school, where study rhythms are structured by periodic exams, assigned tasks, and continuous teacher supervision, university education requires students to self-manage both their learning and the time needed to meet academic deadlines. While this autonomy can foster personal responsibility, it also exposes many students to the risk of procrastination, particularly when they lack effective self-management strategies. This observation is consistent with evidence showing that younger college students procrastinate more than older students [14]. Moreover, systematic reviews and meta-analyses indicate that procrastination tends to decrease with age and that it may decrease over the course of study [4,31,32]. These findings, which can be interpreted in relation to younger people greater sensitivity to delay and immaturity of their self-regulation processes [33,34], also suggest difficulties in perceiving themselves as fully responsible agents in their learning. This occurs during a transition phase intended to facilitate their entry into the professional world and adulthood but often marked by great uncertainty.

University contexts are embedded in societies where many young adults experience a sense of generalized precariousness and perceive that significant effort is required to build their future. This is particularly evident in the Italian context, where youth employment rates are among the lowest in Europe, while the proportions of young people not in employment, education, or training (NEET) and living in households with very low work intensity are among the highest for the 15–29 age group [35]. In addition, there is an increasing perception of uncertainty and pessimism about the future from the beginning of adolescence. Compared to 2021, by 2023 the proportion of young Italians aged 11 to 19 fascinated by the future had decreased by almost 5 percentage points, while the share who are afraid of it had increased by 5.5 percentage points. Moreover, younger adolescents also appear more inclined to remain in Italy as adults: among 11–13-year-olds, 51.4% expect to live in Italy; among 14–16-year-olds, 41.8%; and among 17–19-year-olds, 41.7% [36].

In this context, young adult university students perceive social demands for individual perfection and success, which can influence their tendency to procrastinate [37], as has also been observed in research conducted in other countries [38,39,40]. It should be emphasized that socially prescribed perfectionism (i.e., the perception that others expect one to be perfect) has become a public health concern. Indeed, cultural pressures that promote the pursuit of unattainable perfectionist ideals convey the message that individuals who fail to meet these expectations will suffer consequences [41], such as difficulties entering the job market. These dynamics can influence academic trajectories, which in turn affect educational attainment, widely recognized as a key social determinant of health. In this regard, education strongly shapes employment opportunities and career trajectories, thereby enhancing income levels and long-term economic stability [42,43]. These socioeconomic advantages constitute important pathways linking education to health outcomes. Lower levels of education are consistently associated with poorer health in adulthood, including a higher prevalence of chronic diseases, greater functional limitations, and poorer overall health. Furthermore, the influence of education extends beyond the individual level, as it has been shown that parents’ level of education also affects their children’s health [44].

Consequently, factors that compromise academic path in early adulthood may contribute not only to future socioeconomic disadvantages but also to disparities in physical and mental health. From a public health perspective, understanding the psychological processes that influence students’ academic progress, such as socially prescribed perfectionism and procrastination, may therefore be crucial not only for academic achievement but also for the prevention of long-term social and health inequalities.

In a scenario where real opportunities appear less accessible than in the past, certain aspects of university culture may further amplify an individualistic conception of education and success. Consequently, students may tend to measure their value in terms of performance and merit rather than personal and collective growth, postponing tasks until they are reasonably certain that their abilities will not be challenged.

Given the importance of understanding a temporally oriented behavior in social contexts where urgency and attitudes toward the future seem crucial for young people, qualitative studies appear particularly well-suited to exploring students’ perspectives on how these contexts shape their behavior. In fact, they allow researchers to capture nuances, subjective perceptions, and relational dynamics that are often overlooked in quantitative research. Studies of this type have identified several reasons for procrastination as perceived by university students, emphasizing antecedents such as perfectionism, low motivation, task characteristics, poor regulation, and low external structure [45,46,47].

To our knowledge, in Italy, no qualitative studies have explored the reasons for academic procrastination and the possible role of contextual specificities and institutional features of the university system.

Based on the above, as part of a larger project designed to investigate the tendency to procrastinate in young adulthood, the present study aimed to explore the academic procrastination of Italian university students and the individual, interpersonal, and contextual reasons for procrastination from their perspective.

## 2. Materials and Methods

### 2.1. Participants and Procedure

The participants in the study were initially 99 university students (*Mage* = 21.64; 81% female and 19% male) from the Department of Humanities at the University of Naples Federico II, recruited between May and June 2023 during in-person educational activities. Specifically, they were second-year undergraduate students enrolled in a Bachelor’s degree program in Psychological Sciences and Techniques. Students were informed of the project’s general objectives and invited to participate. Before the data collection phase, an introductory lesson was conducted to provide general information on procrastination and to familiarize students with the topic in broad, non-evaluative terms, drawing on existing theoretical and research literature. At the end of the lesson, interested students were invited to join focus groups of 8–10 participants to further explore their experiences and perceptions. Following this invitation, 10 students withdrew without providing reasons, resulting in a final sample of 89 participants. A total of 10 focus groups were conducted online. Participants were prompted to reflect on whether they recognized themselves in the dynamics of academic procrastination and to discuss the factors that, in their opinion, led them to postpone university-related tasks, providing examples from their personal lives. The discussion guide was not pilot tested prior to data collection.

Each session lasted from 38 to 82 min (mean duration: 61 min), depending on the level of interaction and depth of discussion within the group. The sessions were audio-recorded with participants’ consent and moderated by the first and second authors, each of whom conducted five focus groups. No additional researchers were present during the discussions.

Participants were assigned unique codes to distinguish their contributions during data management. To report the results, these codes are not presented alongside quotations, as the analysis was conducted at the corpus level rather than by individual participant.

Participation in the study was voluntary, anonymous, and unpaid. All subjects gave their consent to participate and to the audio recording of the focus groups. The audio recordings were transcribed verbatim by the first and second authors and checked for accuracy against the original recordings prior to analysis. Adherence to the Consolidated Criteria for Reporting Qualitative Research (COREQ) [48] ensured transparency and methodological rigor in reporting (see Supplementary Materials).

### 2.2. Author Positioning and Reflexivity

The authors, two Italian men and two Italian women, young adults in training and adult university professors, reflected on their positioning regarding the research topics and their relationship with the interviewees.

The first author is a man, a young adult, currently a PhD candidate, who at the time of data collection was a Master’s degree student in Psychology and was exploring the topic of procrastination for his thesis. He had previously completed the same course of study as the interviewees, within the expected timeframe. He was still living with his family of origin.

The second author is a woman, a young adult, currently a PhD in developmental psychology, who at the time of data collection was a doctoral student and a trainee in group psychotherapy. During her studies, she had experienced forms of procrastination, which, however, did not affect the consistently high quality of her results throughout her academic path. At the time of data collection, she was living in an independent home, separate from her family.

The third author is an adult woman, with two young adult daughters still in training, a professor of developmental and educational psychology, and a psychotherapist. She was responsible for teaching a course on certain adolescent issues in which the interviewees had participated and who then voluntarily agreed to join the focus groups. The course was highly interactive, and students were invited to share the difficulties and needs they were experiencing in their university life. This habit of speaking up likely encouraged their participation in research aimed at identifying the factors that hinder their well-being as students.

The last author is an adult man, without children, a professor of dynamic psychology and psychoanalyst, engaged in research and psychotherapy on unconscious dynamics involved in the construction of the self in young adults. The participants had not yet encountered him as their teacher.

Throughout the research process, the authors engaged in reflective discussions about their perspectives and expectations regarding the topics under study, particularly valuing their belonging to the two roles (teachers and trainees) and to the two age categories (young adults and middle adults). The four researchers worked together to minimize the undue influence of their position and subjective experiences through note-taking, discussions, and reflective practices. All authors collaboratively reviewed the international literature, the data analysis procedures, and the interpretation and labeling of the results. A high level of agreement was achieved, and any discrepancies were resolved through collective discussion. Data saturation was reached when no additional themes or insights emerged from the discussions.

### 2.3. Data Analyses

The corpus derived from the focus group transcripts was initially analyzed using Thematic Analysis of Elementary Contexts (TAEC) in T-Lab Plus [49]. The theoretical hypothesis supporting this type of qualitative-quantitative analysis is that, at both the semantic and structural levels, it is possible to identify significant occurrences that reflect the primary ways in which the narrated experience is organized. After a process of disambiguation and lemmatization of words, the TAEC divides the text into elementary context units (ECUs) based on the distribution of words in terms of co-occurrences. It then identifies clusters using an ascending, unsupervised hierarchical method. Each cluster consists of a vocabulary of keywords that appear in specific ECUs, classified by decreasing chi-square value. The clusters are then labeled by the researchers through an interpretative process of the vocabulary and ECUs. In particular, the labelling process involves a detailed examination of each keyword and its placement within the context units by at least two judges.

Starting from the identified clusters, a secondary Reflexive Thematic Analysis [50] was conducted using MAXQDA software (version 26.0, VERBI Software, Berlin, Germany) [51] to explore in depth some of the themes that emerged. The indications provided by Braun and Clarke [52] were followed, including the phases of familiarization, code generation, research, revision, theme definition and naming, and report writing. All researchers independently coded the transcripts, then met to discuss initial codes, reflectively exploring differences in interpretation. No formal assessment of coder agreement was performed, in line with the principles of Reflective Thematic Analysis, which emphasize researcher subjectivity and reflexivity. Themes were developed iteratively through discussion and reflection on the data with all members of the research team.

As a result of this second phase, it was possible to identify further significant themes, consistent with the theoretical and research perspective adopted.

Finally, the encoded data was further explored using MAXQDA’s Code Relations Browser to identify code intersections, i.e., multiple codes applied to the same text segment. Intersections allow researchers to observe how codes combine and co-occur within narratives, revealing connections and patterns across individual, interpersonal, and contextual dimensions. This approach made it possible to identify significant thematic relationships, providing a more articulated representation of the complexity of the reported participants’ experiences and the transversally relevant codes.

## 3. Results

### 3.1. TAEC Clusters Description

The corpus obtained from the transcriptions of the focus groups included 67,967 occurrences, 5456 forms, and 3364 lemmas. Of the 1492 identified ECUs, 1444 were classified (stability index of the analysis: 96.78%) and organized into seven clusters. For each cluster some representative ECUs are reported.

Cluster 1. Exam procrastination as an avoidance strategy

The narratives collected in this cluster illustrate how academic procrastination can serve as a form of avoidance, allowing students to evade confronting their own insecurities and feelings of not being able to tackle study content that may appear difficult to understand, demanding, or burdened by either excessive or insufficient expectations regarding outcomes. One participant, discussing her study habits in demanding courses, clarified:

“I don’t think my procrastination stems from a lack of motivation, because I actually enjoy studying all the subjects I study, but I think I tend to procrastinate because I lack self-confidence.”

Similarly, when describing the moment of beginning a new subject, another student highlighted how anticipated difficulty can generate avoidance:

“I think my procrastination is also due to the fact that, in a certain sense, I don’t want to start studying because I’m afraid that I’ll actually have difficulty understanding the subject matter.”

Cluster 2. Contextual factors facilitating academic procrastination

In this cluster, students suggest that certain elements of university organization contribute to an environment that encourages delaying academic tasks. Among the elements mentioned are the non-mandatory lessons, long journeys, tiring schedules, and the high availability of exam sessions, which lead to perceive postponement as always possible. For instance, while discussing attendance practices, one student explained:

“For example, even attending lectures has sometimes been difficult for me, because, since attendance isn’t mandatory, I’d think, ‘Well, maybe I’ll go tomorrow’, but then tomorrow would come, and I’d say, ‘I’ll go next week’ and so I ended up in a bit of a difficult situation because of that.”

Likewise, reflecting on exam scheduling, another participant noted:

“It becomes much easier to know that there is one date in July, one in September, and one in the extraordinary session to say, ‘Oh well, I’ll do it another time’, and then later, for example, find yourself back at square one, and anyway, later, I don’t know, you start studying the week before or three days before the exam.”

Cluster 3. Individual vs. Contextual sources of procrastination

This cluster gathers reflections on the roots of procrastination. Students question whether their tendency to postpone origins from personal characteristics, such as laziness or difficulties in managing and organizing their study, or whether it arises from contextual factors within the university system. In this movement between individual and situational explanations, students seem to be seeking a clearer understanding of the challenges they face in pursuing their studies. This is evident in a participant’s critical examination of her own explanatory framework, as shown in the following excerpt:

“Perhaps I have too individualistic an approach, in which I see procrastination as closely linked to individual factors such as perfectionism, laziness, and negligence.”

Another student framed this tension as an open analytical question:

“I have put together some questions that, in a hypothetical interview, would ask how much, in our opinion, procrastination stems from an individual component and how much from a structural component, for example, specifically in the university environment.”

Cluster 4. Studying in groups: collaboration, disengagement and competition

In this cluster, the role of the group in the tendency to postpone emerges. Students describe teamwork as an ambivalent experience, capable of both amplifying and mitigating procrastination. In some cases, this dual nature is evident, as illustrated by a participant who emphasizes both the risks of competition and the benefits of group work:

“Maybe, I don’t know, am I less talented than them? It could happen, but I’ll do my best. It’s very important; working in a team helps you develop lots of skills, and even having a group discussion has lots of advantages.”

In addition, students frequently report challenges in group work, particularly the need to take personal responsibility and the risk of distraction caused by social interactions. These concerns are reflected in the following excerpts:

“In a group, you need to have a strong sense of responsibility, because it’s something that actually happened to me in a group I was part of, where one person completely washed their hands of the whole thing.”

“These video calls for studying have been suggested many times in our group, but I’m always a bit reluctant because, I don’t know, it’s a bit dangerous, because I might get distracted, because there might be people in the group who are a bit more fun, and then we start laughing and I get distracted and that’s it, I’m not studying anymore.”

Cluster 5. Pressure and guilt

In this cluster, students discuss how perceived pressure and guilt contribute to procrastination, explaining it in terms of fear of failure. The guilt of not studying enough and postponing exams is described as difficult to overcome. In several discussions, students reported a recurring emotional cycle in which procrastination intensified pressure rather than alleviating it. More participants described this dynamic as an escalating vicious circle:

“Procrastinating, feeling guilty, and feeling guilty because you feel guilty, you start to feel bad until maybe you manage to get out of this endless loop and say, ‘Ok, enough, it’s time to react and go take that damn exam’, then they fail you, amen, you get a grade you don’t like, you accept it, you reject it.”

“You also feel the pressure, you become more anxious and therefore start to feel bad, and this mental factor can also add to your difficulty in taking exams, and so you fail once, you fail twice, this goes on and on, well, endlessly, for a large number of times.”

Cluster 6. “Starting on the right foot”: early experiences and individual academic planning

This cluster collects students’ reflections on how their first experiences at university shape their long-term approach to studying. Specifically, it is emphasized that initial exam results and early experiences can reinforce the tendency to postpone. On the contrary, the ability to plan the path from the beginning and organize the exams can counteract this tendency. One student emphasized the symbolic weight of initial exam results:

“Then something that really influences, in my opinion, the whole university experience is the way you approach exams—it’s even like the first grade you get on your very first exam. For example, if you get, I don’t know, an 18 or a 20, you think, ‘Well, I’m struggling’. But if you get a 30, or a 30 with honors, you think ‘Wow, okay, then…’.”

Another described how early delays can accumulate over time:

“So, you know what happens? For example, you might fall behind on one or two exams, and it throws everything off. I mean, I think that if I had started earlier, maybe… because, you know, in the first semester of my first year, I did three exams instead of four. I know that, maybe, if I had stayed on schedule, I wouldn’t have ended up with that delay, you know?”

Cluster 7. “Does the early bird catch the worm?”: daily study time management

In this cluster, students highlight differences and similarities in personal rhythms, underscoring how study habits affect the tendency to delay.

Some students struggle to start studying in the morning and only find their maximum concentration later in the day:

“Another problem I have is that I also can’t manage to wake up early in the morning. But in general, I don’t really study in the morning anyway, because I procrastinate studying during the day. So, I often end up in the late afternoon, or even in the evening, having to start studying, and I tend to study mostly in the evening, sometimes very late at night.”

Others notice that starting the morning well sets the tone for a productive day:

“In fact, it’s the same for me too. If I start the morning well, I realize that I’ve been productive in the morning, so automatically I can study in the afternoon, and I’m even able to study continuously throughout the day. However, if I get off to a bad start or maybe get up late, so I don’t start studying, automatically I don’t start in the afternoon either.”

### 3.2. Results of the Reflexive Thematic Analysis

This section presents the thematic areas, themes, and codes developed through Reflexive Thematic Analysis, based on the TAEC results, with a specific in-depth analysis of the findings from Cluster 5. The contents of this cluster were considered particularly relevant for the present study, both because they are consistent with the theoretical orientation adopted and because they suggest the relevance of contextual aspects that have been little explored. These aspects have the potential to significantly highlight social and cultural dynamics that can influence academic procrastination and the associated emotional experiences. The analysis, therefore, aimed to deepen the understanding of an emerging thematic area, and the cluster, identified as a broad semantic and conceptual area related to pressure and self-evaluation, was divided into conceptually related categories to demonstrate its specificities

Four thematic areas have been identified, based on 28 codes grouped into 10 overarching themes. The coding tree is presented in Table 1.

The Reflexive Thematic Analysis revealed that procrastination is deeply intertwined with various forms and sources of pressure perceived by university students, which can generate intense, performance-related emotional experiences. In particular, the results show that the tendency to postpone assumes specific functions in relation to the management of pressure, the experience of time, and especially the future, as well as high self-expectations and their social and institutional production.

#### 3.2.1. The Functions of Procrastination: Seeking and Avoiding Pressure

A first thematic area concerns the functions attributed to procrastination, which emerges as an ambivalent mode of interacting with pressure. On the one hand, postponing is described as an intentional study strategy, on the other as a way of shirking responsibility, capable of mitigating the weight of commitment and the risk of failure.

Some participants reported that experiences of procrastination have taught them that delaying academic tasks allows them to work better, since approaching deadlines enables them to be more focused and discover personal skills they were not aware of, as one student clearly explained:

“Sometimes I procrastinate in the sense that... I even complain when I find myself having to do everything at the last minute, but it’s as if I unconsciously want to. It’s as if that pressure to do everything at the last minute gives me that extra push to say: ‘I have to make it’. It’s like it gives me more energy. Then I get there, maybe I complain, cry, have a meltdown... but it’s as if I wanted it.”

This sense of efficiency under pressure is further emphasized by the fact the performance outcomes are comparable to those achieved if one had started earlier:

“And that’s precisely the advantage, that’s the advantage. The fact that you get the same results as if you started studying two or three months earlier, you get the same results as two or three weeks earlier. Got it?”

In this sense, procrastination is not only sought but also portrayed as an effective study method, capable of producing satisfactory results despite less time invested. Participants note, however, that while this is productive, it also causes tiredness and fatigue.

At the same time, procrastination assumes a defensive function. Postponing allows students to avoid full responsibility for either the commitment or the outcome. In the event of failure, not having studied in time becomes a plausible explanation that protects self-worth, functioning as a form of salvific self-sabotage, as illustrated by the following quotation:

“In my experience, I tend to delay starting to study for a given exam because, well, as I mentioned before, in a way this allows me to justify it if things go badly. I mean, somewhat consciously and unconsciously, I know that, for example, working under pressure is not good for me—not because I can’t focus, but because at some point I burn out, there’s too much going on, and I can’t manage myself. And why do I delay when I know I feel this way? Because at worst, I can justify myself if the exam doesn’t go as I expect. Basically, in my experience, this is the explanation I’ve given myself for procrastinating when it comes to studying.”

Some students also described procrastination as a way to avoid confronting commitments, for instance by postponing the request for the final thesis to a professor, so as not to make the project of completing studies real:

“Even though I’m in my second year and I know very well that there are some professors with whom I’ve thought about writing my thesis, and even though I more or less already know the topic and know I have to contact them because they’re very in-demand professors, I just don’t do it because doing it would make me responsible for the request. So, just to avoid taking responsibility and making the matter too real or serious, I tend to procrastinate.”

In both situations, procrastination allows individuals to maintain distance from the weight of responsibility, reducing the pressure to face it immediately.

#### 3.2.2. Social Clock Pressure and Perceptions of the Future

A second thematic area regards the pressure related to social clock time and, in particular, to the future. Participants frequently reported anxiety and uncertainty about what awaits them, a threatening future, and a labor market they perceive as unstable and unpredictable. The future is described with concern, and the present is characterized by feelings of suspension and difficulties in making decisions for one’s own path. As the participants highlighted:

“In fact, this is why I said that it is the fear of the future that could lead university students to procrastinate, since let’s say, not everyone, but maybe, like in my case, when I am studying, I am not working, so practically my parents support me. Whereas from the moment I graduate, it’s normal that my parents expect me to make my own project, my own future and to support myself, so, let’s say, procrastinating and not taking exams, staying at university, is kind of a cradle, quote unquote.”

“I think that procrastination is often due to a fear of the future, because often, especially at our university, we’re told that it’s difficult to find a job, and so maybe a person tends not to take exams precisely in order to avoid graduating and then find themselves without a job.”

In this context, socially shared temporal norms help define the “right” timing for completing studies, transitioning to subsequent stages of life, and reaching adulthood. Recurring questions, such as “When will you graduate?”, suggest normative pressure, highlighting fears of falling behind peers or failing to meet social and familial expectations, as noted by more students:

“From my peers, I don’t really feel that much pressure, like, you know, the ‘you have to rush, you have to rush’. But it’s also true that, let’s say, more on the micro level of the university context, I don’t feel that much pressure. If we look at the macro level, though, a bit yes, you do feel the pressure of being told ‘you have to graduate on time’, because this or that person got recognition for graduating within a certain amount of time...”

“When I hear that other people are already getting interested in so many things, I always feel behind. I say to myself, ‘I have no interests’, like ‘what am I even doing with my life’, but maybe I’m giving importance to other things, and maybe we don’t really give weight to that.”

Within this thematic area, one participant also addressed procrastination as a mechanism of temporal stasis, imagining it as a behavior potentially signaling his need for personal time.

“Procrastination seems like postponing and not having any more time, whereas maybe postponing is useful because you are not wasting that time, you are giving it to yourself.”

#### 3.2.3. Internal Pressure and Emotional Responses

Another thematic area concerns internally perceived pressure and the emotional experiences associated with academic performance. For instance, students describe studying as an unavoidable obligation, an activity perceived as pervasive and necessary in one’s life, in the face of which one must give up something else. As one participant explained:

“Sometimes I realize that I see university as the thing, the only most important thing I’m doing in my life, and so I think I should maybe give more, or study more, invest more time, or maybe sometimes I also feel wrong when I’m not giving the right importance to certain things.”

This perception contributes to a sense of constraint that, according to students, makes everything outside studying seem like a waste of time. Alongside this, the idea that “it’s never enough” emerges strongly: many participants report setting very high standards for themselves, feeling the need to do more than is required, often exceeding what is necessary for exams:

“Many times, we can’t really explain the reason why we tend to procrastinate, maybe because we don’t recognize the fact that we’re seeking this kind of perfectionism. We might not acknowledge it, but it’s part of us… so you’re never really satisfied with what you’ve learned, because you end up thinking that there are actually so many things to know, so many things you could add, so many things the professor might ask, and you then tend to add a lot of things that might actually be superfluous and not even required.”

Preparation is always perceived as incomplete, and the fear of failure reinforces this perception. One participant described this fear as closely tied to personal ambitions and comparisons with others:

“…Yes, in fact, the question of ‘I like what I study’ is maybe precisely because I like what I study and I want to succeed in my future job… I limit myself because I’m afraid of failing in what I love and of not succeeding compared to others who studied with me and who may, quote unquote, surpass me…”

These internal standards are accompanied by intense emotional experiences related to performance, including feelings of inadequacy. A student, reflecting on her experiences during her first year at university, explained:

“Some (exams) scared me a little, others I considered too difficult, beyond my reach. This pressure only fueled this sense of inadequacy, and so it was the session in which I procrastinated the most. In other words, I basically only took one exam in the summer session, and I took all the others later.”

Another highlighted the guilt that often accompanies last-minute work:

“I always end up putting off studying for exams until the last minute, literally the very last minute, crying, screaming, and wanting to tear my hair out, and feeling very guilty too, because I realize that, objectively, I could manage my time much better and that it’s my own fault I find myself in these situations.”

Some students also described feeling stuck, as if excessive pressure and expectations prevent action, thereby contributing to procrastination:

“It is with university that the fear of making mistakes and falling behind completely paralyses me and therefore causes me to find myself in an extreme situation, namely the simple fact that thinking that my actions today could affect my future and what my life will be like leads me to procrastinate a lot, because I want to put off this decision more and more so that I don’t have to take responsibility for it.”

#### 3.2.4. Social and Institutional Drivers of Pressure

A final thematic area concerns the perception of social and institutional reinforcement of the pressure students experience. At the cultural level, references to a performance culture emerge, supported by communication about successful models in the media and on social networks, perceived as unattainable:

“Society puts this idea into our heads: ‘Okay, you go to university, you do a bachelor’s degree, in three years you have to graduate, then you do a master’s, and after five years you have to immediately find a job’… all this social pressure, and who end up deciding to kill themselves, or to lie to their parents.”

“If I’m not mistaken, a girl was praised so much because she graduated at 23, with five degrees, three master’s degrees... It is also communication, how things are communicated, that leads individuals to perceive this anxiety, this failure, and therefore, in my opinion, procrastination itself.”

These dynamics both favor and reflect the internalization of high expectations, contributing to the perception that students always need to meet standards, which students describe as part of their “genetic makeup”. As one participant noted while reflecting on academic procrastination:

“…Because at some point we are so influenced by our parents, society, etc., that we internalize this idea that we must achieve high grades, otherwise it’s as if we weren’t good enough. It’s a bit of a strange cycle, because it has its advantages and disadvantages. When you get a 30, you say ‘Wow, I’m good’ but when you get a lower grade or fail, your world falls apart.”

Peer comparisons also intensify these dynamics:

“I’m going to take the exam with a classmate of mine, and when we get different grades… I feel like I’m inferior to her if she gets a slightly higher grade than me. So, let’s say that you always tend to measure yourself against everyone, not only your parents, friends, etc., but also strangers.”

At the institutional level, critical issues related to the organization of the university exam calendar and the quality of education were reported, underscoring a misalignment between the values the university declares and its actual practices:

“Another thing I always thought about regarding the organization of the university and exams is how this acceleration of time damages the actual quality of study… This is something I often wonder about, especially when I talk to my classmates and they tell me that they prepared for their exams using summaries or studied from the professors’ notes, so I say to myself: ‘Yes, I’m on schedule, but what’s the point? Why pay university fees, why spend time at university if this is what studying is like?’. Sometimes I think that maybe I could go a little faster, I could take all my exams in this summer session, have a quiet summer and study from the transcripts and summaries, and I did... I had a quiet summer, but then what will I actually have left of these studies in 20 years?”

“Society, even though it wants to communicate positive messages like ‘everyone should do things in their own time’, all these positive messages ultimately push for perfectionism… many master’s programs award points based on how long it took you to graduate.”

As one student highlighted, positive guidance from professors, however, can mitigate this pressure:

“When a professor tells you that time isn’t important, as Professor P. did, for example, it doesn’t matter how long it takes you to graduate, it doesn’t matter what your grades say about you… I would be more relaxed, I believe.”

Finally, the family represents another relevant context. Parental expectations, a sense of responsibility or guilt, and intra-family comparisons all contribute to reinforcing the weight of academic experience. Participants described feeling accountable not only to themselves but also to their parents:

“Actually, last summer, when I found myself a bit struggling with the last two exams I had to take and I saw my dad, I was already warning him, like, ‘Dad, look, in October we have to pay the first installment’ you know? I felt guilty because I thought ‘Okay, he’s not making me work, he lets me study quietly at home, he makes sacrifices for everything’, I mean, you even feel guilty because you think ‘Wow, these people are paying for my university, I absolutely cannot go beyond the normal time’.”

In some cases, the pressure appears to lead students to deny or hide their academic difficulties, while in other cases, a sense of indebtedness emerges:

“I also feel a lot of pressure from, for example, my mother… and this actually leads me to lie to her sometimes, for example on days when I’m at home alone and she comes back and asks ‘Did you study today?’, I say ‘Yes’ when in reality I haven’t studied all day.”

“Sometimes we don’t just want to fulfill our own expectations, because we all hope to achieve certain goals, but we also want to fulfill the goals, or desires, of other people, such as our parents in this case, even though they may not put pressure on us, even though, at least in my case, they don’t get involved in these matters, which obviously concern only me, but it’s something I feel I have to do anyway, to give them some satisfaction too.”

Others mentioned comparisons within the family as an added source of pressure:

“I am the first granddaughter of my grandmother to go to university, although I’m not the first to go, and all my relatives have completed their university studies with very high grades… this perhaps influences me in accepting a certain grade, and therefore, if I have to reject that grade, it’s an exam that I have to retake, when my goal in the end was just to graduate.”

### 3.3. Code and Thematic Intersections

The intersections among codes, analyzed using MAXQDA’s Code Relations Browser, enabled the identification of relationships within and across thematic areas. The graphs, generated using the software’s graphical visualization tools and presented in the figures below, constitute an exploratory map of the intersections. The thickness of the lines indicates the greater recurrence whereby specific dimensions co-emerge in the participants’ narratives and, together with the number of connected codes, suggest possible nodes of interpretative interest.

**Figure 1:**
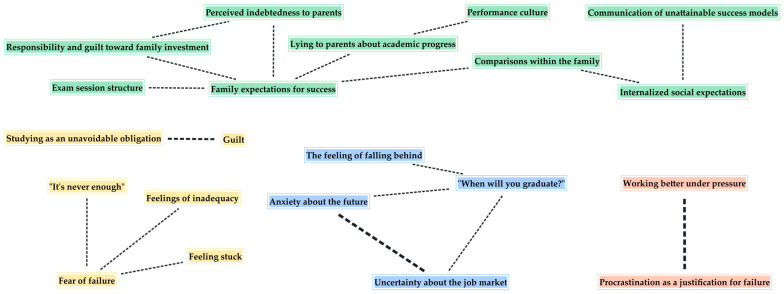
Code intersections within thematic areas.

Figure 1 shows the intersections among codes belonging to the same thematic area. Codes that have not shown any co-occurrence—“Same results in less time”, “Procrastination as granting oneself time”, “Concerns about the quality of education”, “The positive role of professors”, “Misalignment between declared values and university practices”, “Peer comparison” and “Avoiding commitment through postponement”—are excluded from the graphical representation.

**Figure 2:**
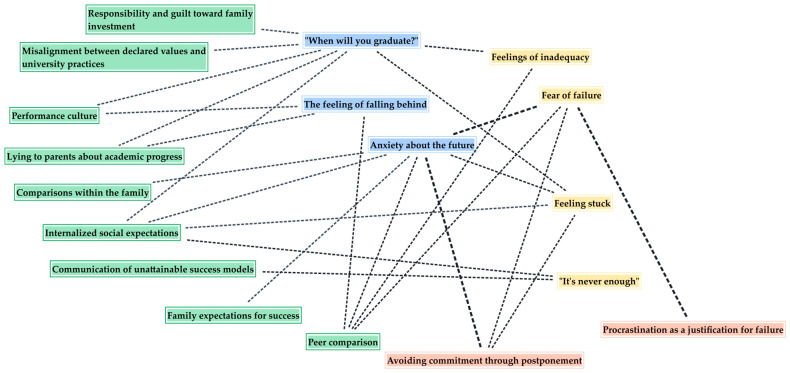
Code intersections across thematic areas.

Figure 2 shows the intersections among codes from different thematic areas. Codes that have not shown any co-occurrence with codes outside the same area—“Working better under pressure”, “Uncertainty about the job market”, “Studying as an unavoidable obligation”, “Guilt”, “Perceived indebtedness to parents”, “Exam session structure”, “Same results in less time”, “Procrastination as granting oneself time”, “Concerns about the quality of education” and “The positive role of professors”—are excluded from the graphical representation.

As shown by the maps, some codes display a higher number of transversal intersections than others, thereby positioning themselves as central nodes within the relational network. In particular, “Anxiety about the future” emerges as a highly connective code, being the one that co-occurs most frequently with those belonging to different thematic areas. In addition to repeatedly co-occurring with “Uncertainty about the job market”, it intersects with family and peer comparisons, internalized and familial expectations, and experiences of fear of failure and postponed commitment. Alongside “Feeling stuck” and “Feelings of inadequacy”, it is one of the only codes present across all thematic areas, suggesting these serve as points of convergence in participants’ narratives.

Another central code is “When will you graduate?”, which co-occurs with all the codes belonging to its thematic area and with several others representing familial, social and institutional sources of pressure, as well as performance-related emotional states. Among these, students’ fear of failure appears alongside feelings of inadequacy, feeling stuck, and a sense that their efforts are never enough, highlighting their tendency to coexist in academic procrastination, while the perception that studying is an unavoidable obligation co-occurs solely with guilt, indicating the interplay of obligation and self-critical emotions.

Regarding the functions of procrastination, students’ tendency to procrastinate to work better under pressure appears uniquely alongside the use of procrastination to justify failure, which repeatedly co-occurs with fear of failure. By contrast, avoiding commitment through postponement appears in connection to different emotional experiences, such as anxiety about the future, fear of failure and feelings of being stuck.

Other notable co-occurrences include “The feeling of falling behind”, which intersects with performance culture, peer comparison, and lying to parents about academic progress, and the pairing of “It’s never enough” with internalized expectations and unattainable success models.

Social and cultural pressure-related codes co-occur with each other and connect with codes reflecting temporal pressure and internal emotional experiences. Notably, internalized social expectations are particularly salient, appearing alongside temporal pressures related to graduation, anxiety about the future, feeling stuck, and the perception that one must always do more.

Overall, a configuration of interrelated experiences emerges, with certain codes functioning as core aspects that bridge emotional, social, and temporal dimensions of academic pressure.

## 4. Discussion

The current study aimed to explore the perceptions of young Italian university students regarding academic procrastination, using a qualitative design with a two-stage analysis. This enabled, on the one hand, a broad exploration of university students’ representations and narratives on academic procrastination and, on the other hand, a focused in-depth examination of a thematic area that emerged as particularly interesting. An initial Thematic Analysis of Elementary Contexts served an exploratory function, identifying clusters that reflect the complexity and multidimensionality of the phenomenon, while the subsequent Reflexive Thematic Analysis provided a more detailed articulation of the dynamics related to perceived pressure and students’ emotional experiences.

The results of the exploratory phase confirm the evidence in the literature on the multifactorial nature of procrastination, arising from interactions among individual, emotional, relational, and environmental factors [4,19,47,53]. The clusters identified describe procrastination as a form of task avoidance (Cluster 1), as the result of a university organization that makes postponement structurally possible or even probable (Cluster 2), as an object of questioning and attribution of meaning between individual and contextual explanations (Cluster 3), as an ambivalent experience mediated by comparison with peers (Cluster 4), as closely intertwined with external pressure and internal guilt (Cluster 5), as a process that develops over time but starting from early academic experiences (Cluster 6), and, finally, as influenced by personal rhythms and study habits that are not always functional (Cluster 7).

In general, the thematic groups that emerged are consistent with the results and developments in international research on procrastination. It is widely recognized that procrastination can be used to avoid a task, which can cause doubts and insecurities about one’s abilities and may therefore need to be addressed in the short term [11,54]. In addition, studies focusing on social and organizational aspects have emphasized the role of the group and the characteristics of university organization. It has been repeatedly suggested that group work would positively affect task persistence [55], reducing the tendency to postpone [47,56]. However, in line with the results of the present qualitative study, which show how the group can be both a resource and a risk factor for procrastination, Svartdal et al. [28] observed that group work can reduce procrastination when it is characterized by the feeling of interdependence among members, which the university system often does not favor, encouraging individualistic and competitive tendencies. Moreover, university students often struggle to work effectively in groups, which can promote widespread irresponsibility.

Referring to the organization of the institution, the lack of external structuring by the university, reflected in the excessive freedom students perceive in organizing their studies, which was absent in their previous school experience, is instead unanimously regarded as a risk factor [28,46,47,57]. For instance, ample time to complete a task can alter perceived difficulty, making it appear particularly complex through the so-called “mere deadline effect” [58].

Clusters 6 and 7 are aligned with research showing that students who report stronger time-management skills are less inclined to postpone [59]. This finding is consistent with evidence that planning, time management, self-regulation, and goal clarity are some of the main protective factors against academic procrastination [7,47].

Finally, it is noteworthy that Cluster 3, which pertains to doubts and reflections regarding the individual or contextual nature of procrastination, appears to mirror trends observed in the scientific literature, where the focus has progressively shifted from a purely individual perspective on procrastination to a more social and relational one, encompassing broader and more complex levels of analysis [27,28]. Just as research highlights the interplay between individual characteristics, task-related factors, and the social context, students’ narratives similarly suggest that the tendency to postpone cannot be fully understood by separating personal factors from the environmental conditions in which studying takes place, yielding a thematic cluster characterized precisely by such reflections.

Within this broad framework, Cluster 5 is salient for the centrality of experiences of pressure and guilt, emerging as an area of particular interest that condenses references to perceived pressures and to emotional and self-evaluation experiences. In research on procrastination, several studies have focused on the relationship between procrastination and guilt [54,60] and specific forms of pressure, such as time pressure (i.e., the subjective perception of having less time than necessary to complete a task or achieve a goal) [61]. The observation that these two themes co-occur in this cluster raises interesting questions. For this reason, we further explored the thematic cluster, investigating how pressure was understood in relation to procrastination.

Secondary thematic analysis showed that procrastination assumes different functions in relation to pressure. In line with the findings of the exploratory phase, procrastination is described both as a strategy for avoiding negative emotions that may arise from failure and as a means of enhancing performance in academic tasks, creating a state of activation that enables optimal results under pressure. Narratives that describe studying after procrastination as a ‘mystical’ experience that leads to the rediscovery of incredible study skills refer to a phenomenon similar to *pressurization* [28], and *arousal procrastination* [62], both of which describe how postponing study can enhance productivity. This ambivalence is articulated in this analysis by showing how procrastination can simultaneously support performance and protect self-worth, as highlighted by the analysis of the intersections within the thematic area of the functions of procrastination. This appears to be relevant in university contexts, where failure is experienced as particularly threatening, and may help explain why procrastination is so widespread among young people and student populations [2,4,5,6,7,8,47]. However, it is noteworthy that, from the participants’ perspective, procrastination appears to function simultaneously as a virtuous cycle that can enhance productivity—shaping a specific approach to studying—and as a vicious cycle, from which it is difficult to escape, since the conditions in which studying occurs are often marked by discomfort and disrupted sleep due to the limited time available to achieve the desired outcomes. This distinction is central to avoiding conflating the students’ descriptions of a form of procrastination that functions productively with the concept of active procrastination proposed by Chun Chu and Choi [63].

A relevant contribution of the in-depth analysis concerns the role of the temporal dimension and the future. Time-related pressure emerges not only in relation to deadline management, as time pressure [64], but as a broader experience, in which the future is perceived as uncertain, unstable, and full of expectations.

The co-occurrences among concerns about the future, keeping pace with others, and meeting normative timelines, on one hand, and expectations related to peers, family, and broader society, on the other, suggest that the experience of time—particularly the future—interacts with standards, norms, and ideals of success that shape students’ expectations regarding what they should achieve and by when. Indeed, although economic and social changes support an extended transition into adulthood, an ideology persists that assumes the attainment of adulthood within timelines that are no longer adequate [65], thereby reinforcing feelings of inadequacy when falling behind. In this regard, the study by Culatta and Clay-Warner [66] is particularly relevant, showing that parental, societal, and peer expectations regarding the timing of various markers of adulthood have a substantial effect that can override the weight of individual expectations on anxiety and depression in young adults.

The intersection of anxiety about the future with the fear of failure and the use of procrastination as a way to avoid the formalization of commitments then assumes particular importance, if considered in the light of the developmental tasks of the young adult, including entering the world of work, building relational networks, and negotiating one’s role in society. Social temporal norms seem to function as pressure devices that link the present act of studying to the possibility of future realization, against which procrastination may represent a form of temporal suspension, allowing the delay of confronting decisions and steps that could be experienced as decisive for “growing up”. In this sense, the question “When will you graduate?” serves as a catalyst for external pressures and internalized expectations about becoming “performing adults”. This perspective seems to be in line with the intimate connection, underlined in the literature, between the perception of social expectations of perfection and worries and anxieties about the future [41].

Furthermore, the intersections within and across themes related to social sources and internal experiences of pressure suggest a close connection among cultural, institutional, and family expectations in shaping university students’ life experiences. Familial expectations for success appear to be connected both to elements of responsibility and guilt (“Responsibility and guilt toward family investment”, “Perceived indebtedness to parents”), and to dysfunctional practices of pressure management (“Lying to parents about academic progress”). These connections show the link between external demands and morally binding imperatives, to which students may feel obliged to respond. The co-occurrences with “Comparisons within the family” appear to further indicate an evaluative context within the family that reinforces these expectations. Additionally, intersections between “Performance culture” and “Lying to parents about academic progress”, and between “Communication of unattainable success models” and “Internalized social expectations” highlight how these family dynamics are situated within a broader social framework defined by high and often unattainable models of success, suggesting that procrastination may function as a response to demands perceived as excessive or contradictory, among which the most illustrative is the expectation to be both perfect and fast at the same time.

The inextricable link across the various sources of pressure is consistent with the concept of the “generalized other” [67]. Indeed, performance expectations are not attributed exclusively to specific individuals but are also expressed as widespread, impersonal, and collective social norms. This helps explain why participants sometimes do not identify a specific actor as the source of perceived social pressure, instead referring to an abstract “other” who embodies socially shared values of success, achievement, and merit.

In light of these observations, the tendency to procrastinate on academic tasks emerges as an experience strongly characterized by moral and self-evaluative dimensions, in which studying is sometimes perceived as a pervasive obligation to be fulfilled in order for one’s value to be recognized by others, the violation of which may cause a considerable sense of guilt, as suggested by the intersections observed.

Taken together, these findings indicate that academic procrastination should be understood as a behavior rooted in contemporary temporality and society, shaped by specific cultural and institutional contexts that influence the university experience of young adults and must be carefully considered.

### Strengths, Limitations, and Future Research Directions

This qualitative study enabled an in-depth exploration of participants’ perceptions, opinions, and experiences, yielding rich, multifaceted testimonies on academic procrastination in young adulthood. The interaction among focus group members encouraged the emergence of social dynamics and both shared and divergent viewpoints, prompting reflection on issues often examined from an individual perspective. This approach, therefore, contributed to a more accurate, contextualized, and complex understanding of the phenomenon under study. Furthermore, while previous research has increasingly considered social factors in academic procrastination, this attention has largely focused on material aspects directly related to studying, such as task characteristics or group work. This study suggests that the perspective should be extended to include broader sociocultural influences transmitted through educational institutions and mass media, particularly expectations regarding academic performance and deadlines. These not only shape student behavior, often leading to maladaptive coping strategies such as procrastination, but also influence personality factors, including perfectionism, that are commonly implicated in procrastination tendencies.

However, the research has some limitations. The results cannot be generalized to larger populations, both because of the small sample size and its predominantly female composition. In addition, the presence of the facilitator and group dynamics may have introduced influence and social desirability biases. Moreover, participants were recruited from students enrolled in the same degree program within the Department of Humanities, in the context of their educational activities, which may have introduced selection bias and further limited the generalizability of the findings.

In the future, it would be desirable to expand and diversify the focus groups to include participants from fields outside the humanities, thereby increasing the transferability of the results and deepening the validity of the thematic categories that emerged from larger, more gender-balanced samples. The integration of quantitative approaches or mixed methods could also help strengthen the robustness of the findings, allowing for a more comprehensive assessment of the phenomenon under investigation.

## 5. Conclusions

The qualitative research presented in this study aimed to provide an articulated account of academic procrastination from the perspective of Italian university students, highlighting its profoundly multidimensional nature and its entrenchment in the social, institutional, and cultural contexts in which young adults’ university experiences take shape. Through two phases of analysis, the study explored procrastination within the university environment, underscoring the significant role of personal, organizational, relational, and emotional factors. In particular, an in-depth examination of experiences of pressure and guilt revealed the central role of temporal and success-related expectations—both internalized and socially shared—in shaping students’ tendency to delay and their relationship with the future. From this perspective, procrastination can be interpreted as a possible response to the normative demands for efficiency, speed, and perfection that characterize contemporary society and the university system, which appears to function as a sounding board for a performative culture, offering a possible temporal coping strategy in relation to life transitions perceived as highly evaluative and oriented toward an uncertain future. Beyond its psychological impact, delays in academic progress can lead to longer time to degree, higher dropout rates, and reduced academic achievement, with downstream consequences for workforce readiness and the timely development of skilled professionals in critical sectors such as healthcare, education, and public service. These delays can translate into reduced lifetime earnings, delayed economic independence, and lower cumulative tax contributions, generating measurable economic costs to society. Furthermore, if maladaptive delay patterns persist into professional life, they can affect productivity, organizational efficiency, and long-term planning capabilities within institutions. Taken together, these behavioral and economic consequences position academic procrastination as a phenomenon with clear public health relevance, warranting preventive and systemic interventions that go beyond the individual student and address institutional practices, resource allocation, and early mental health support strategies. In this vein, the findings suggest the importance of university interventions and policies that, in addition to promoting individual skills in planning and time management, address the depth of the roots of procrastination. For instance, group counseling sessions could be promoted, focusing on perfectionism, perceived social pressure, and uncertainty about the future, offering students a safe space for discussion and sharing. Recognizing that these experiences are not isolated individual experiences, but widely shared in the academic context, can help reduce feelings of inadequacy and self-blame. For this to occur, higher education institutions must pay greater attention to the sociocultural dimensions that shape the experience of academic procrastination and critically examine how current educational contexts influence the messages young adults receive from society.

## Figures and Tables

**Table 1:** Table of thematic areas, themes and codes.

Thematic Area	Theme	Code (Frequency)
The functions of procrastination: seeking and avoiding pressure	Delaying as an effective study strategy	Working better under pressure (8)
		Same results in less time (1)
	Postponing responsibility through delay	Procrastination as a justification for failure (7)
		Avoiding commitment through postponement (3)
Social clock pressure and perceptions of the future	Future-related anxiety and uncertainty	Anxiety about the future (9)
		Uncertainty about the job market (9)
	Temporal norms and life-course expectations	“When will you graduate?” (15)
		The feeling of falling behind (4)
	Procrastination as temporal suspension	Procrastination as granting oneself time (2)
Internal pressure and emotional responses	Internal standards and self-demands	“It’s never enough” (7)
		Studying as an unavoidable obligation (3)
	Performance-related emotional experiences	Fear of failure (11)
		Feelings of inadequacy (6)
		Guilt (4)
		Feeling stuck (3)
Social and institutional drivers of pressure	Sociocultural pressure	Internalized social expectations (13)
		Peer comparison (4)
		Communication of unattainable success models (3)
		Performance culture (3)
	Institutional pressure	Misalignment between declared values and university practices (2)
		Concerns about the quality of education (1)
		Exam session structure (1)
		The positive role of professors (1)
	Family pressure	Family expectations for success (8)
		Responsibility and guilt toward family investment (9)
		Lying to parents about academic progress (2)
		Comparisons within the family (2)
		Perceived indebtedness to parents (2)

## Data Availability

The data presented in this study are available upon request from the corresponding author.

## Supplementary Materials

The following supporting information can be downloaded at: https://www.mdpi.com/article/10.3390/ijerph23030374/s1, The COREQ Checklist. Reference [48] is cited in the Supplementary Materials.

## Abbreviations

The following abbreviations are used in this manuscript:

COREQ	Consolidated Criteria for Reporting Qualitative Research
TAEC	Thematic Analysis of Elementary Contexts
ECU	Elementary Context Unit

## References

[1] Yan B., Zhang X. (2022). What Research Has Been Conducted on Procrastination? Evidence From a Systematical Bibliometric Analysis. Front. Psychol..

[2] Balkis M., Duru E., Bulus M. (2013). Analysis of the Relation between Academic Procrastination, Academic Rational/Irrational Beliefs, Time Preferences to Study for Exams, and Academic Achievement: A Structural Model. Eur. J. Psychol. Educ..

[3] Klingsieck K.B. (2013). Procrastination: When Good Things Don’t Come to Those Who Wait. Eur. Psychol..

[4] Steel P. (2007). The Nature of Procrastination: A Meta-Analytic and Theoretical Review of Quintessential Self-Regulatory Failure. Psychol. Bull..

[5] Onwuegbuzie A.J., Jiao Q.G. (2000). I’ll Go to the Library Later: The Relationship between Academic Procrastination and Library Anxiety. Coll. Res. Libr..

[6] Prohaska V., Morrill P., Atiles I., Perez A. (2000). Academic Procrastination by Nontraditional Students. J. Soc. Behav. Personal..

[7] Klassen R.M., Ang R.P., Chong W.H., Krawchuk L.L., Huan V.S., Wong I.Y.F., Yeo L.S. (2010). Academic Procrastination in Two Settings: Motivation Correlates, Behavioral Patterns, and Negative Impact of Procrastination in Canada and Singapore. Appl. Psychol..

[8] Ozer B.U., Demir A., Ferrari J.R. (2009). Exploring Academic Procrastination among Turkish Students: Possible Gender Differences in Prevalence and Reasons. J. Soc. Psychol..

[9] Senécal C., Julien E., Guay F. (2003). Role Conflict and Academic Procrastination: A Self-determination Perspective. Eur. J. Soc. Psychol..

[10] Ferrari J.R., Pychyl T.A. (2012). “If i Wait, My Partner Will Do It”: The Role of Conscientiousness as a Mediator in the Relation of Academic Procrastination and Perceived Social Loafing. N. Am. J. Psychol..

[11] Sirois F., Pychyl T. (2013). Procrastination and the Priority of Short-Term Mood Regulation: Consequences for Future Self. Soc. Personal. Psychol. Compass.

[12] Salvatori C. (2017). Se non ora quando? Procrastinazione: Origine e trattamento. Cogn. Clin..

[13] Wäschle K., Allgaier A., Lachner A., Fink S., Nückles M. (2014). Procrastination and self-efficacy: Tracing vicious and virtuous circles in self-regulated learning. Learn. Instr..

[14] Kim K.R., Seo E.H. (2015). The Relationship between Procrastination and Academic Performance: A Meta-Analysis. Pers. Individ. Differ..

[15] Kim S., Fernandez S., Terrier L. (2017). Procrastination, Personality Traits, and Academic Performance: When Active and Passive Procrastination Tell a Different Story. Pers. Individ. Differ..

[16] Batool S.S. (2020). Academic achievement: Interplay of positive parenting, self-esteem, and academic procrastination. Aust. J. Psychol..

[17] Klassen R.M., Krawchuk L.L., Rajani S. (2008). Academic Procrastination of Undergraduates: Low Self-Efficacy to Self-Regulate Predicts Higher Levels of Procrastination. Contemp. Educ. Psychol..

[18] Goroshit M., Hen M. (2021). Academic Procrastination and Academic Performance: Do Learning Disabilities Matter?. Curr. Psychol..

[19] Sirois F.M., Pychyl T.A. (2016). Procrastination, Health, and Well-Being.

[20] Anwar T., Sitwat A. (2024). Perfectionism, Academic Procrastination and Psychological Distress in University Students. Clin. Couns. Psychol. Rev..

[21] Argiropoulou M., Vlachopanou P. (2022). The Role of Psychological Distress as a Potential Route Through Which Procrastination May Confer Risk for Reduced Life Satisfaction. Curr. Psychol..

[22] Constantin K., English M.M., Mazmanian D. (2018). Anxiety, Depression, and Procrastination among Students: Rumination Plays a Larger Mediating Role than Worry. J. Ration.-Emotive Cogn.-Behav. Ther..

[23] Stöber J., Joormann J. (2001). Worry, Procrastination, and Perfectionism: Differentiating Amount of Worry, Pathological Worry, Anxiety, and Depression. Cogn. Ther. Res..

[24] Beutel M.E., Klein E.M., Aufenanger S., Brähler E., Dreier M., Müller K.W., Quiring O., Reinecke L., Schmutzer G., Stark B. (2016). Procrastination, distress and life satisfaction across the age range—A German representative community study. PLoS ONE.

[25] Demir S., Kuşcu Karatepe H. (2025). The Effect of Academic Procrastination on Life Satisfaction Among Nursing and Midwifery Students: The Serial Mediation Role of Academic Self-Efficacy and Self-Control. Behav. Sci..

[26] Steel P., Klingsieck K.B. (2016). Academic Procrastination: Psychological Antecedents Revisited. Aust. Psychol..

[27] Nordby K., Klingsieck K.B., Svartdal F. (2017). Do Procrastination-Friendly Environments Make Students Delay Unnecessarily?. Soc. Psychol. Educ..

[28] Svartdal F., Dahl T.I., Gamst-Klaussen T., Koppenborg M., Klingsieck K.B. (2020). How Study Environments Foster Academic Procrastination: Overview and Recommendations. Front. Psychol..

[29] Serrano Corkin D.M., Lindt S.F., Williams P.S. (2021). Effects of Positive College Classroom Motivational Environments on Procrastination and Achievement. Learn. Environ. Res..

[30] Patrzek J., Grunschel C., Fries S. (2012). Academic Procrastination: The Perspective of University Counsellors. Int. J. Adv. Couns..

[31] Rodríguez A., Clariana M. (2017). Procrastinación en Estudiantes Universitarios: Su Relación con la Edad y el Curso Académico. Rev. Colomb. Psicol..

[32] van Eerde W. (2003). A meta-analytically derived nomological network of procrastination. Pers. Individ. Differ..

[33] Ferrari J.R. (1991). Self-Handicapping by Procrastinators: Protecting Self-Esteem, Social-Esteem, or Both?. J. Res. Pers..

[34] Steinberg L., Graham S., O’Brien L., Woolard J., Cauffman E., Banich M. (2009). Age Differences in Future Orientation and Delay Discounting. Child. Dev..

[35] European Commission/EACEA (2025). The Situation of Young People in the European Union.

[36] ISTAT (2024). Indagine Bambini e Ragazzi—Anno 2023. Nuove Generazioni Sempre più Digitali e Multiculturali.

[37] Sommantico M., Postiglione J., Fenizia E., Parrello S. (2024). Procrastination, Perfectionism, Narcissistic Vulnerability, and Psychological Well-Being in Young Adults: An Italian Study. Int. J. Environ. Res. Public Health.

[38] Closson L.M., Boutilier R.R. (2017). Perfectionism, Academic Engagement, and Procrastination among Undergraduates: The Moderating Role of Honors Student Status. Learn. Individ. Differ..

[39] Kathleen E., Yulianti D. (2021). The Relationship Between Perfectionism and Academic Procrastination in College Students Learning Online Due to the COVID-19 Pandemic. Proceedings of the International Conference on Economics, Business, Social, and Humanities.

[40] Onwuegbuzie A.J. (2000). Academic Procrastinators and Perfectionistic Tendencies among Graduate Students. J. Soc. Behav. Personal..

[41] Flett G.L., Hewitt P.L., Nepon T., Sherry S.B., Smith M. (2022). The Destructiveness and Public Health Significance of Socially Prescribed Perfectionism: A Review, Analysis, and Conceptual Extension. Clin. Psychol. Rev..

[42] Carlson R., McChesney C. (2015). Income sustainability through educational attainment. Exchange.

[43] Pensiero N., Barone C. (2024). Parental Schooling, Educational Attainment, Skills, and Earnings: A Trend Analysis across Fifteen Countries. Soc. Forces.

[44] Suiter S.V., Meadows M.L. (2023). Educational Attainment and Educational Contexts as Social Determinants of Health. Prim. Care.

[45] Abramowski A. (2018). Is Procrastination All That “Bad”? A Qualitative Study of Academic Procrastination and Self-Worth in Postgraduate University Students. J. Prev. Interv. Community.

[46] Grunschel C., Patrzek J., Fries S. (2013). Exploring Reasons and Consequences of Academic Procrastination: An Interview Study. Eur. J. Psychol. Educ..

[47] Klingsieck K.B., Grund A., Schmid S., Fries S. (2013). Why Students Procrastinate: A Qualitative Approach. J. Coll. Stud. Dev..

[48] Tong A., Sainsbury P., Craig J. (2007). Consolidated criteria for reporting qualitative research (COREQ): A 32-item checklist for interviews and focus groups. Int. J. Qual. Health Care.

[49] Lancia F. (2012). The Logic of the T-LAB Tools Explained.

[50] Braun V., Clarke V. (2019). Reflecting on Reflexive Thematic Analysis. Qual. Res. Sport Exerc. Health.

[51] VERBI Software (2025). MAXQDA Software.

[52] Braun V., Clarke V. (2006). Using Thematic Analysis in Psychology. Qual. Res. Psychol..

[53] Karimi Moonaghi H., Baloochi Beydokhti T. (2017). Academic Procrastination and Its Characteristics: A Narrative Review. FMEJ.

[54] Fee R.L., Tangney J.P. (2000). Procrastination: A Means of Avoiding Shame or Guilt?. J. Soc. Behav. Personal..

[55] Johnson D.W., Johnson R.T. (2009). An educational psychology success story: Social interdependence theory and cooperative learning. Educ. Res..

[56] Koppenborg M., Klingsieck K.B., Hüffmeier J. (2024). Conjunctive and Additive Group Work Reduce Academic Procrastination: Insights from a Vignette Study. Curr. Psychol..

[57] Parrello S. (2026). Giovani adulti e malattia d’idealità: Procrastinazione dei compiti accademici o evolutivi?. Riv. Polisanalisi.

[58] Zhu M., Bagchi R., Hock S.J. (2019). The mere deadline effect: Why more time might sabotage goal pursuit. J. Consum. Res..

[59] Limone P., Sinatra M., Ceglie F., Monacis L. (2020). Examining Procrastination among University Students through the Lens of the Self-Regulated Learning Model. Behav. Sci..

[60] Giguère B., Sirois F.M., Vaswani M., Sirois F.M., Pychyl T.A. (2016). Delaying things and feeling bad about it? A norm-based approach to procrastination. Procrastination, Health, and Well-Being.

[61] Kühnel J., Bledow R., Kuonath A. (2023). Overcoming procrastination: Time pressure and positive affect as compensatory routes to action. J. Bus. Psychol..

[62] Ferrari J.R. (1992). Psychometric Validation of Two Procrastination Inventories for Adults: Arousal and Avoidance Measures. J. Psychopathol. Behav. Assess..

[63] Chun Chu A.H., Choi J.N. (2005). Rethinking Procrastination: Positive Effects of “Active” Procrastination Behavior on Attitudes and Performance. J. Soc. Psychol..

[64] Szollos A. (2009). Toward a Psychology of Chronic Time Pressure: Conceptual and Methodological Review. Time Soc..

[65] Fingerman K.L. (2017). Millennials and Their Parents: Implications of the New Young Adulthood for Midlife Adults. Innov. Aging.

[66] Culatta E., Clay-Warner J. (2021). Falling Behind and Feeling Bad: Unmet Expectations and Mental Health during the Transition to Adulthood. Soc. Ment. Health.

[67] Mead G.H. (1934). Mind, Self and Society from the Standpoint of a Social Behaviorist.

